# COVID-19 and its genomic variants: Molecular pathogenesis and therapeutic interventions

**DOI:** 10.17179/excli2022-5315

**Published:** 2022-09-13

**Authors:** Pulak R. Manna, Zackery C. Gray, Malabika Sikdar, P. Hemachandra Reddy

**Affiliations:** 1 Department of Internal Medicine, Texas Tech University Health Sciences Center, School of Medicine, Lubbock, TX 79430, USA; 2 Department of Zoology, Dr. Hari Singh Gour Vishwavidyalaya, Sagar, MP 470003, India; 3 Department of Pharmacology and Neuroscience, Texas Tech University Health Sciences Center, Lubbock, TX 79430, USA; 4 Neurology, Departments of School of Medicine, Texas Tech University Health Sciences Center, Lubbock, TX 79430, USA; 5 Public Health Department of the Graduate School of Biomedical Sciences, Texas Tech University Health Sciences Center, Lubbock, TX 79430, USA; 6 Department of Speech, Language and Hearing Sciences, School Health Professions, Texas Tech University Health Sciences Center, Lubbock, TX 79430, USA; 7 Nutritional Sciences Department, College of Human Sciences, Texas Tech University, Lubbock, TX 79409, USA

**Keywords:** COVID-19 and its variants, pathogenesis, healthy immunity, lifestyle, immunocompromised conditions, therapeutics

## Abstract

Coronavirus disease-19 (COVID-19), caused by a β-coronavirus and its genomic variants, is associated with substantial morbidities and mortalities globally. The COVID-19 virus and its genomic variants enter host cells upon binding to the angiotensin converting enzyme 2 receptors that are expressed in a variety of tissues, but predominantly in the lungs, heart, and blood vessels. Patients afflicted with COVID-19 may be asymptomatic or present with critical symptoms possibly due to diverse lifestyles, immune responses, aging, and underlying medical conditions. Geriatric populations, especially men in comparison to women, with immunocompromised conditions, are most vulnerable to severe COVID-19 associated infections, complications, and mortalities. Notably, whereas immunomodulation, involving nutritional consumption, is essential to protecting an individual from COVID-19, immunosuppression is detrimental to a person with this aggressive disease. As such, immune health is inversely correlated to COVID-19 severity and resulting consequences. Advances in genomic and proteomic technologies have helped us to understand the molecular events underlying symptomatology, transmission and, pathogenesis of COVID-19 and its genomic variants. Accordingly, there has been development of a variety of therapeutic interventions, ranging from mask wearing to vaccination to medication. This review summarizes the current understanding of molecular pathogenesis of COVID-19, effects of comorbidities on COVID-19, and prospective therapeutic strategies for the prevention and treatment of this contagious disease.

## Introduction

COVID-19, a very infectious disease, is caused by severe acute respiratory syndrome coronavirus 2 that was first identified in December 2019 at Wuhan, China (Berekaa, 2021[12]; Liu et al., 2020[89]). This virus has since spread globally, leading to a severe health crisis. COVID-19 is a member of the family of viruses known as *Coronaviridae* (order, *Nidovirales*; subfamily, *Orthocoronavirinae*), which displays similar clinical, as well as pathological, features as those of SARS (Severe Acute Respiratory Syndrome) and MERS (Middle East Respiratory Syndrome) (Liu et al., 2020[89]; Sheervalilou et al., 2020[133]; Umakanthan et al., 2020[153]). Airborne transmission is the primary mode of infection in the spread of the COVID-19 virus and its variants, and enters host cells upon binding to the angiotensin converting enzyme 2 (ACE2) receptor that is expressed in a variety of tissues (Rando et al., 2021[119]; Yuki et al., 2020[174]). The pathophysiological manifestations of COVID-19 include moderate to life-threatening symptoms that are frequently associated with fever, headache, respiratory distress, hypoxia, lung injury, inflammation, and cardiovascular diseases (CVDs). COVID-19 can lead to grave outcomes by affecting the immune system and damaging multiple organ systems through a plethora of pathophysiological events (Hu et al., 2021[70]; Ye et al., 2020[171]). 

A large body of epidemiological evidence indicates a strong correlation between the intake of vitamins, minerals, antioxidants, and a reduction and/or prevention of COVID-19 and other pathogens (Manna et al., 2016[98], 2022[95]; Pecora et al., 2020[113]). Nutritional status can influence COVID-19 infections to variable degrees, with implications for duration, harshness, and overall consequences. Deficiency of nutrients, involving impaired immunity, leaves individuals more susceptible to severe COVID-19 related infections and fatal outcomes (Calder, 2020[22]; Gorji and Khaleghi Ghadiri, 2021[59]). Nevertheless, people with immunocompromised conditions are more inclined to develop multi organ complications and deadly consequences from COVID-19 (Kalra et al., 2021[78]; Mainali and Darsie, 2021[94]).

COVID-19 placed an immense burden on nearly every country in the world. A wide variety of measures to control this virus were implemented including: lockdowns, social distancing, mask wearing, vaccinations, and many emergency use authorization (EUA) drugs and/or antibodies (Bhatti et al., 2020[14]; Kandimalla et al., 2020[80]; Manna et al., 2022[95]). However, genetic variants of the disease have prolonged the fight against COVID-19 and pushed healthcare systems to their limits. Herein, we summarize current literatures that help comprehend the molecular pathogenesis of the disease, its relevance to healthy immunity, and therapeutic potentials for the management of COVID-19. 

## COVID-19: Its Emergence and Pathophysiological Consequences

As stated above, COVID-19 began as global concern in late 2019 and early 2020 beginning in Wuhan, China and spreading throughout the world. COVID-19 is an acute respiratory disease that attacks alveolar epithelial cells of the lungs, with clinical features essentially similar to SARS and MERS, which are spherical (60-200 nm) and single stranded RNA viruses (Berekaa, 2021[12]; Umakanthan et al., 2020[153]). Stemming from the same family of coronaviruses, COVID-19 was thought to emerge as a zoonotic transmission from bats, being the major evolutionary reservoirs of coronavirus diversity historically. Upon emergence, this virus made human to human transmission and spread rapidly throughout the globe with a high morbidity and mortality (Sheervalilou et al., 2020[133]; Umakanthan et al., 2020[153]). The World Health Organization (WHO) declared COVID-19 a global pandemic in March 2020. While the majority of COVID-19 patients display mild to moderate symptoms, a small number of cases develop critical signs, including pneumonia, ARDS (acute respiratory distress syndrome), sepsis, multi organ failure, and death (Rong et al., 2021[122]; Xu et al., 2020[168]). Of note, the median incubation period for COVID-19 was estimated to be between 5 and 6 days. Despite various pathophysiological conditions, COVID-19 associated infections and mortalities are considerably higher among men in comparison to women (Manna et al., 2022[95]). 

COVID-19 is spread from human to human through both direct contact and airborne transmission, including coughing, sneezing, or normal breathing. Various activities and social settings have different tendencies to spread the virus and present varied levels of risk. A wide variety of risk factors for COVID-19 include activities, procedures, products, and events, ranging from low to very high (Liu et al., 2020[89]; Manna et al., 2022[95]). Noteworthy, however, are the contributions of numerous risk factors to COVID-19 infections and resultant complications that depend on immune health, aging, and underlying medical conditions. There is increasing evidence that children and adolescents are generally asymptomatic to COVID-19, or exhibit mild symptoms such as fever, headache, fatigue, and nasal congestion, then recover from the infection by their healthy immune system (Lu et al., 2020[90]; Rando et al., 2021[119]). Conversely, a subset of patients, possessing impaired immunity or immunocompromised conditions, display severe clinical manifestations, requiring hospitalizations and life supporting treatments, along with mortalities.

**Figure 1:**
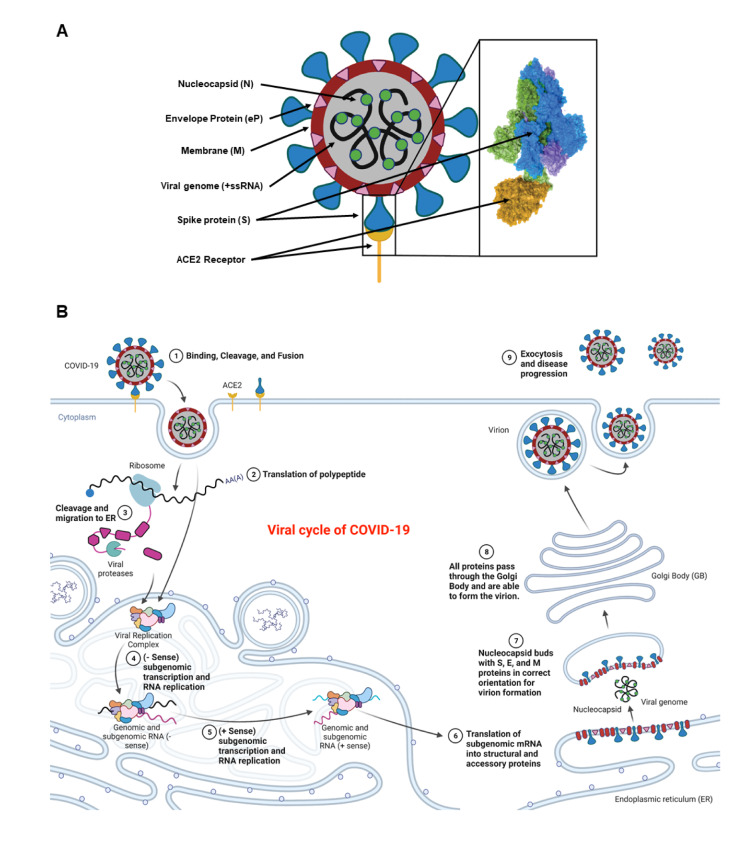
Schematic representation of a COVID-19 virus and its different components including the spike protein (blue), plasma membrane (red circle), nucleocapsid proteins (green circles), positive sense single stranded RNA (black lines), ACE2 receptor (yellow umbrella-shaped), and the envelope protein (pink triangles). The spike protein is further magnified to show various parts including the receptor binding pocket and its binding to the ACE2 receptor (yellow) (A). Entry of the COVID-19 virus into a body cell upon binding, cleavage, and fusion to the ACE2 receptors. Initial +ssRNA is translated using host cellular machinery to make the viral replication complex within the host endoplasmic reticulum (ER). This complex is then used in conjunction with the +ssRNA to make -ssRNA copies that allow for genomic duplication. These duplicates are then translated to make all the virus specific proteins. The host ER then positions all viral proteins for subsequent migration to the Golgi Body (GB). As the vesicle is moved to the GB, it engulfs a copy of the +ssRNA viral genome and forms a virion (B). The virion is migrated to the plasma membrane where it is expulsed and can now infect new somatic cells or cells of another individual. Retrieved and revised from https://app.biorender.com.

The genomic configuration of COVID-19 is highly conserved with previously identified SARS and MERS, all of which possess large positive sense RNA (+RNA) genomes. Among the four different isoforms (α, β, γ, and δ), COVID-19 is categorized as a β-virus that enters host cells through endocytosis involving three steps: binding, cleavage, and fusion. It encompasses four structural proteins; spike (S), membrane (M), envelope protein (eP), and nucleocapsid (N) (Figure 1A(Fig. 1)). The spike protein is composed of two functional subunits, S1 and S2, in which the former binds to the ACE2 receptors (Manna et al., 2022[95]; Rando et al., 2021[119]). The binding of S2 allows for insertion of the RNA genome into the host cells, which then undergoes cleavages by host proteases (e.g. furin and trypsin), and translation to form polyproteins that are then assembled to make replication-transcription complexes. Once the complex is formed, a copy of the RNA genome is made, different structural proteins are synthesized in the cytoplasm, and all parts are assembled with help from the endoplasmic reticulum (ER) and Golgi apparatus (Boopathi et al., 2021[19]; Mir et al., 2021[102]; Rando et al., 2021[119]). These viral particles are then released from the cell by exocytosis and have the ability to infect other cells to continue the replication process. 

The life cycle of the COVID-19 virus (similar to its genomic variants) consists of most of the same steps known to occur to +RNA reproductive cycles (Figure 1B(Fig. 1)). The spike proteins allow for the initial binding and entry of the virus into the host organism. The +RNA genome is then used to synthesize the viral replication complex, which is then put back through the replication complex to create copies of the genome and various respective viral proteins. Notably, the RNA replication requires the synthesis of a -RNA strand to compliment the +RNA strand that will be made and then protein synthesis and RNA replication occurs within the ER. When the proper components become available, the ER buds off to encompass the RNA surrounding it with a membrane containing embedded viral particles. Final post-transcriptional processing occurs within the Golgi apparatus. The completed particle is then free to preform exocytosis and leave the host cell to go infect another host cell. The viral cycle starts upon exposure of COVID-19 and/or its variants, they replicate and spread exponentially in the body, infect others, and eventually die in two weeks. 

## COVID-19 and its Variants, and their Impact on Global Health Tragedy

All COVID-19 variants are single stranded positive sense RNA (+RNA) viruses with roughly ~30 kDa of highly conserved genomic sequences as those of their SARS and MERS counterparts. The WHO has declared certain strains of COVID-19 as Variants of Concerns (VOCs). These variants have increased COVID-19 transmissibility, severity, epidemiology, clinical disease presentation, or have decreased the effectiveness of current treatment options (Alkhatib et al., 2021[4]; Zella et al., 2021[175]). Notably, COVID-19 α, β, γ, δ, and Omicron variants have shown significant effects in different parts of the world, in which both δ and Omicron variants are responsible for the majority of COVID-19 associated complications and mortalities (Alkhatib et al., 2021[4]).

The COVID-19 α-variant (B.1.1.7) was first discovered in September 2020, in the United Kingdom, and was initially considered to have higher rates of transmissibility than the original COVID-19 virus, with the most notable mutation being a N501Y (Asparagine to Tyrosine substitution) and a deletion of amino acid 69/70 on the spike protein causing increased binding affinity (Galloway et al., 2021[54]). It was shown to have a 43-90 % higher transmission rate than the original virus in England but did not increase disease severity (Davies et al., 2021[40]).

The β-variant of COVID-19 (B.1.351) was initially identified in October 2020, in South Africa, and contains the same N501Y mutation along with many others but lacks the 69/70 deletion (Tegally et al., 2021[149]). This variant is not associated with increased transmission or disease severity but showed resistance to neutralizing antibodies due to changes of the spike protein's primary structure, thus, demonstrated possibilities for increased reinfection rates and certain treatment resistance. 

Brazil was thought to be the initial location (November 2020) of the COVID-19 γ-variant (P.1) and consists of the same N501Y spike protein mutation without the 69/70 deletion, but with additional mutations on other components such as the nucleocapsid protein (Gutierrez et al., 2021[63]). The P.1 strain was the most common strain in Brazil during a significant COVID-19 outbreak showing greater disease severity against younger populations, as compared with a previous COVID-19 outbreak mostly consisting of other variants (Freitas et al., 2021[53]). It is thought that mutations to the spike protein account for its ability to evade certain monoclonal antibody treatments (Wang et al., 2021[162]).

The COVID-19 δ-variant (B.1.617.2) was the most predominant strain causing the disease in the United States and accounted for the most cases in the U.S. back in August of 2021 (Jhun et al., 2021[76]; Shiehzadegan et al., 2021[134]). It was first identified in May 2021, in India, and shows many novel mutations such as L452R (Leucine to Arginine) and P681R (Proline to Arginine) of the spike protein that can inhibit antibody binding and increase its affinity for the ACE2 receptor expressed in somatic cells. The δ-variant has a higher transmissibility rate (40-60 % increase over α-variant) and has alluded to more severe complications, including hospitalizations and mortalities, than that of the original COVID-19 virus (Ong et al., 2022[106]; Shiehzadegan et al., 2021[134]).

The Omicron variant (B.1.1.529) is thought to have originated in South Africa, Botswana, or Hong Kong (Callaway, 2021[23]). This COVID-19 variant was first reported in November 2021 and shows signs of considerable reinfection capability, increased transmissibility, disease severity, and augmented antibody evasion (Baker and Van Noorden, 2021[10]). This variant has extensive spike protein mutations, including N501Y and an EPE (Glutamate, Proline, Glutamate) insertion at amino acid 214, nucleocapsid mutations, and other deletions similar to previous VOCs (Callaway, 2021[23]). While genomic and proteomic analyses have identified more than 30 mutations in Omicron from the original COVID-19 virus, much is unknown surrounding this variant (Baker and Van Noorden, 2021[10]). 

Recently, BA.4 and BA.5 have become the newest omicron subvariants to bring cause for alarm. They have been found to evade antibodies produced for past Omicron variants and are being closely monitored as they continue to infect more individuals (Cao et al., 2022[26]). The BA.4 and BA.5 variants are comparable in the spike protein to the BA.2 omicron variant with a 69-70 deletion, L452R (leucine to arginine at 452), F486V (phenylalanine to valine at 486), and amino acid at glutamine 493 (Tegally et al., 2022[148]). It is thought that these variants arose in South Africa and accounted for close to 50 % of the COVID-19 cases in April 2022.

**Figure 2:**
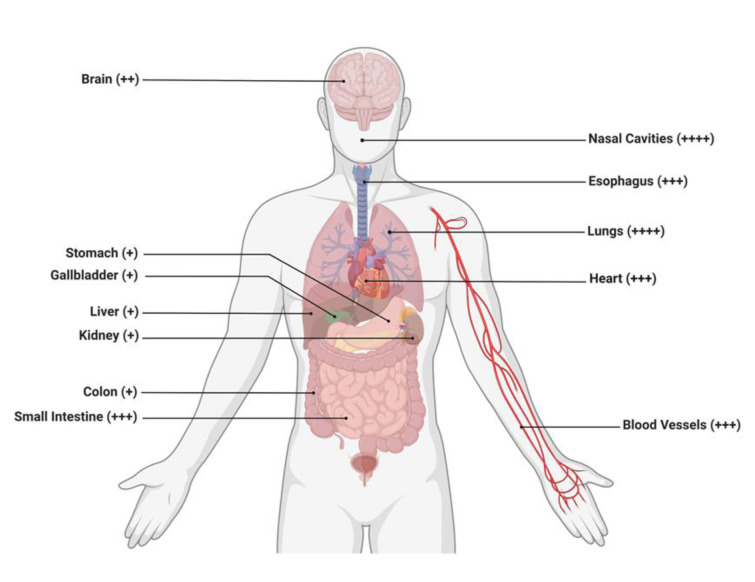
Schematic representation revealing the potential target of the COVID-19 virus or its variants to various organs expressing the ACE2 receptors. The organ/organ systems shown are the brain, stomach, gallbladder, liver, kidney, colon, small intestines, nasal cavities, esophagus, lungs, heart, and blood vessels. Different organs with the ACE2 receptor expression are arbitrarily depicted as + (low), ++ (medium), and +++/++++ (high), thus, demonstrating the diverse ability of these viruses to infect respective organs and result in clinical negative outcomes for the patient. Retrieved and revised from https://app.biorender.com.

It is noteworthy that COVID-19 and its genomic variants enter host cells upon binding to the ACE2 receptors expressed in a variety of tissues, but higher prevalence of this receptor are within the lungs, heart, and kidneys (Figure 2(Fig. 2)).

## Nutrients and Immune Health, and their Relevance to COVID-19

Nutrients are required for normal growth, reproduction, and various physiological activities. Vitamins, minerals, and antioxidants are fundamental to the proper functioning of the immune system, which essentially prevents an organism from contracting COVID-19 and other pathogens. Deficiency of nutrients, involving impaired immunity, is associated with a variety of health issues and increased susceptibility to severe COVID-19 associated complications and mortalities (Calder, 2020[22]; Gorji and Khaleghi Ghadiri, 2021[59]; Jayawardena and Misra, 2020[74]). 

It is unquestionable that nutritional status plays a pivotal role in maintaining bodily homeostasis, as well as overall healthy physiology. Specifically, vitamins are divided into two groups: water-soluble (C and eight B vitamins, i.e., B1, B2, B3, B5, B6, B7, B9, and B12) and fat-soluble (A, D, E, and K). All of these vitamins are obtained from various food sources that people consume regularly, and they exert diverse effects on biological activities. The recommended daily amount (RDA) varies between adult women and men (Table 1(Tab. 1)). B vitamins play integral roles in many important processes in the body such as immune cell proliferation, hormonal equilibrium, energy production, heart and neurological health, oxygen transportation, decreasing the risk of comorbidities, cytokine formation, and antibody production (Manna et al., 2022[95]). These processes help strengthen the immune system for the recognition and neutralization of COVID-19 and other harmful environmental factors. Vitamins A, C, D, E, and K serve in many cellular functions, including anti-inflammation, antioxidation, immunomodulation, and anti-thrombotic states. These diverse processes aid in the de-escalation or protection from severe inflammation and tissue damage inflicted by COVID-19. 

Macronutrients such as carbohydrates, fats, and proteins, provide various building blocks and energy sources for an organism to develop and/or repair immune system function. Certain fats such as omega-3 fatty acids provide anti-inflammatory effects, contribute to appropriate immune responses, and have shown to assist patients with COVID-19 infections and complications (Doaei et al., 2021[43]; Manna et al., 2022[95]).

In addition to macronutrients, various micronutrients are instrumental to the appropriate functioning of the immune system and, therefore, prevent individuals from COVID-19 and other relevant diseases. In accordance, zinc, iron, selenium, copper, and magnesium are essential for the inhibition of viral replication, proliferation of various immune cells and components, anti-inflammation, antioxidation, immunomodulation, and serve as cofactors with enzymes in many necessary reactions (Akhtar et al., 2021[1]).

Anti-inflammatory substances have the ability to assist and/or regulate the body's natural immune response associated with the cytokine storm and influenced by severe COVID-19 infections that cause low oxygen saturation, lung damage, multi organ failure, and ultimately death. Antioxidants provide safe mechanisms to avoid tissue damage and provide an avenue to neutralize reactive oxygen species (ROS) used throughout the immune system. The correct usage of inflammation and ROS provide the immune system with an accurate and efficient response to pathogens, thus increasing chances of survival with minimal damage. Therefore, nutrients, by strengthening and modulating the immune system, serve as the primary defense against COVID-19 and other invading pathogens. 

The immune system is a collection of biological processes, including various organs and cellular structures that prevent organisms from being affected by a variety of environmental toxins, bacteria, and viruses (Chaplin, 2010[27]; Parkin and Cohen, 2001[111]). Briefly, the immune system, involving innate and adaptive/acquired responses, is vital to proper functioning of many important physiological processes, as it serves as a barrier between pathogens and the internal milieu (Hoebe et al., 2004[67]; Tomar and De, 2014[151]). Noteworthy, healthy immunity has recently been reported as a high priority of preventive medicine for combating COVID-19 (Manna et al., 2022[95]). The innate immune system is the culmination of physical barriers and literal gene expression of an organism that is present at birth such as skin, epithelial tissue linings, the respiratory tract, and the genitourinary tract; as well as mucus layers that coat these tissues. Cells and other components, specific to the innate system, are the neutrophils, monocytes, macrophages, cytokines, and specific proteins (such as antimicrobial peptides) that work to broadly attack pathogens and invaders (Beutler, 2004[13]; Niyonsaba et al., 2017[104]). Neutrophils are the body's first cellular line of defense for external pathogens which are ingested through phagocytosis and subsequently metabolized (Beutler, 2004[13]). The adaptive immune system is thought to have evolved alongside the innate system in complex vertebrates to identify and recognize explicit threats to an organism. Both T- and B-lymphocytes generated in the thymus and bone morrow comprise the cellular components of the adaptive immune system, in which mature T-cells are responsible for cytokine production, antigen destruction, and immunomodulation (Bonilla and Oettgen, 2010[18]; Tomar and De, 2014[151]). B-cells recognize a pathogen and develop antibodies against it, which then respond rapidly to recognize and contain infections (Dong, 2021[44]; Hillion et al., 2020[66]; Seifert and Kuppers, 2016[130]). 

**Figure 3:**
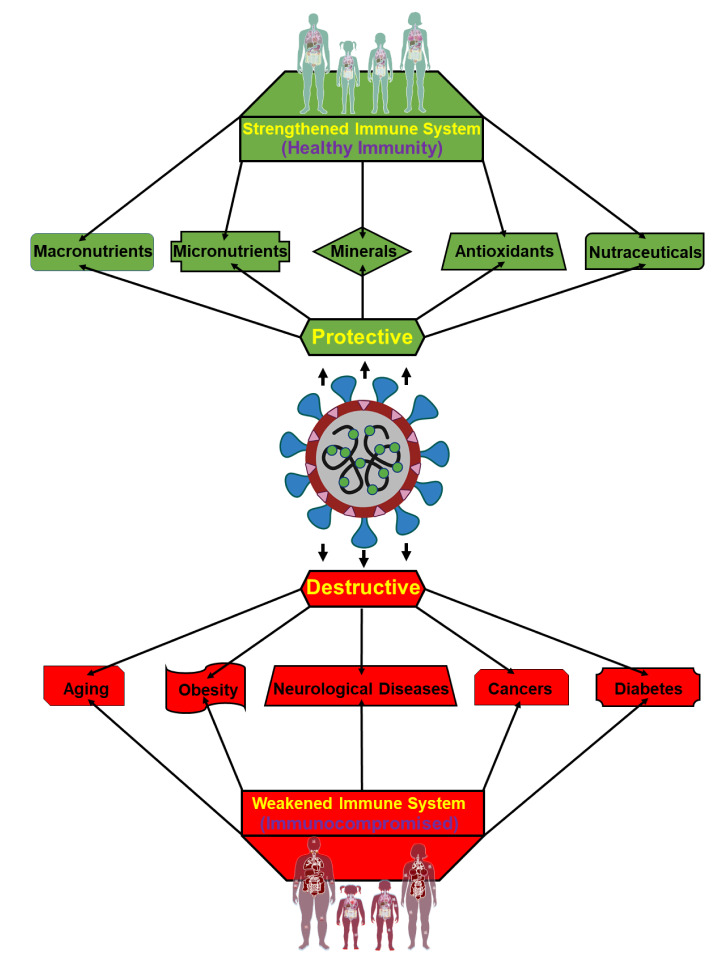
Graphical representation illustrating the impact of COVID-19 to men and women possessing either a strengthened immune system or an impaired immune system. Individuals maintaining healthy immunity/immunomodulation (upper section), effected by vitamins, minerals, and nutrients, are generally protective against COVID-19 (middle) linked infections and resulting consequences. On the other hand, people with immunocompromised conditions (lower section), affected by underlying medical situations, including aging, obesity, cancers, neurological disease, and diabetes are most vulnerable to severe COVID-19 associated infections and complications, with fatal outcomes.

An effective immune response provides its host with the greatest possible chance to weather and protect against various diseases including COVID-19. Whereas immunomodulation, influenced by a variety of health promoting factors including nutrients, contributes to protecting an organism from pathogens and overactive immune responses where immunosuppression is unable to adequately recognize and neutralize those invaders (Figure 3(Fig. 3)). COVID-19 is non-severe among young and healthy, however, it has shown severe effects among a subset of the population such as elderly, obese individuals, and people with other underlying medical conditions (Wolff et al., 2021[167]). These conditions range from such underlying complications to the inadequate intake of nutrients and cofactors. The ability of an organism possessing impaired immunity to fend off infection is especially relevant within the confines of COVID-19. 

## Lifestyle and its Correlation to COVID-19

**Figure 4:**
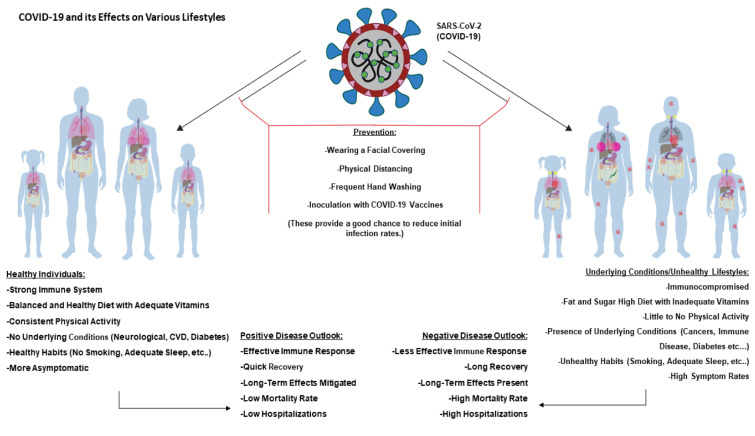
Diagrammatic representation of COVID-19 acting on various lifestyles. Left panel shows healthy male/female and child/adult. Due to their healthy lifestyle choices, adequate intake of nutrients, consistent physical activity, and no underlying conditions, they can present a positive disease outlook. This indicates that the majority of those with healthy lifestyles will muster effective immune responses, quick recoveries, low mortalities, and few hospitalizations. Conversely, right panels show unhealthy individuals presenting opposite conditions as those of healthy individuals. They will majorly experience less effective immune responses, longer recovery times, more long-term effects, higher mortalities, and more hospitalizations. However, both groups can greatly benefit from prevention techniques including wearing facial coverings, physical distancing, adequate handwashing, and receiving a COVID-19 vaccine.

A lifestyle centers around the way an individual lives, and it can be broadly divided into two categories, healthy and unhealthy. While the former lifestyle involves high physical activity, balanced nutrition, and provides a boosted immune system, an unhealthy lifestyle is characterized by low physical activity, unhealthy habits such as smoking and poor nutrition, and leads to an impaired immune system with associated complications and diseases. Both lifestyles are coordinately associated with a number of factors (de Frel et al., 2020[41]; Holly et al., 2020[68]; Li et al., 2018[86]). These factors exert both positive (healthy) and negative (unhealthy) effects on the immune system, reflecting diverse influences on a body's defense mechanisms against various invaders, including COVID-19 and other pathogens (Figure 4(Fig. 4)). 

### Healthy lifestyle

A well-balanced diet, including vitamins, micronutrients, and antioxidants, is vital to maintaining a healthy immune system, as well as many physiological activities. Both balanced diets and active lifestyles are instrumental in the prevention of COVID-19 infections and other relevant diseases (Dean et al., 2022[42]). In fact, the maintenance of a strengthened immune system requires a healthy lifestyle. Nonetheless, physical activity is associated with improved immunosurveillance and immunocompetence as well as mediating pro-inflammatory responses (Fedele et al., 2021[50]; Scheffer and Latini, 2020[128]). In accordance with this, exercise is inversely linked to insulin sensitivity and increases an individual's ability to uptake glucose from the blood. It is also helpful in stress management which is a known influencer of immune health (Borghouts and Keizer, 2000[20]). Exercise boosts the production of endorphins that are beneficial in coping with stress and pain. Mental well-being and stress management can also be maintained with various contemplative and mindfulness activities including yoga, meditation, and nature centric pursuits (Walsh, 2011[160]). Conspicuously, a healthy lifestyle helps maintain bodily homeostasis and is tightly associated with the appropriate functioning of various biological activities including hormone regulation and oxidative stress management (Figure 4(Fig. 4)). In addition to being a direct influence of the innate immune system's ability to combat infection, ROS can result in the destruction of surrounding cells and tissues. This is especially important for aging populations as high ROS is a predominant risk factor in developing CVDs following vascular endothelial degradation (El Assar et al., 2013[46]; Iddir et al., 2020[72]). Overall, a healthy lifestyle is the principle driving force for the maintenance of the boosted immune system that protects individuals from COVID-19 and other invading pathogens. 

### Unhealthy lifestyle

Unhealthy lifestyles include activities, habits, and diets that impair the immune system. For example, unhealthy diets involve elevated levels of sugar, fats, LDLs, and are low in vitamins, nutrients, and antioxidants. These can hinder immune system function and result in many health complications including a higher risk of COVID-19 infections with worse outcomes (Chung et al., 2021[32]; Clemente-Suarez et al., 2021[35]). Unhealthy foods also show a significant correlation to weight gain and obesity leading to chronic pro-inflammatory states. The risk for certain immunosuppressant conditions and diseases such as diabetes and CVDs are also increased. Such conditions along with others naturally predispose individuals to severe COVID-19 infections and outcomes (Elizabeth et al., 2020[48]; Mozaffarian et al., 2011[103]).

Other unhealthy lifestyle choices include excessive alcohol consumption, drug usage, and smoking. Alcohol affects the lungs and their ability to fight off respiratory infections due to suppressed ciliary movements as well as increased overall oxidative stress. Chronic alcohol intake has been shown to create a pro-inflammatory state, hinder the function of immune cells (e.g. macrophages), and affect toll-like receptors through ligand binding inhibition (Yeligar et al., 2016[172]). The proinflammatory state found during a COVID-19 infection is only exacerbated in the presence of alcoholism. Furthermore, substance abuse also incites immunosuppression and has been associated with many serious complications such as CVDs, asthma, cancers, and brain injury. Smoking is detrimental to the immune system and brings about numerous complications, including lung cancers and CVDs. Of note, ~15 % of global death is in some way attributed to the first- or second-hand smoking of tobacco products (Qiu et al., 2017[116]). Dysregulation of the immune system results in inadequate responses to pathogens, increased pulmonary inflammation through T-helper cell promotion and pathogenic severity, and limited overall immune responses to combat infection. The pro-inflammatory state induced by smoking could increase inflammation during COVID-19 cytokine storm, and smoking is detrimental to the lung health resulting in infections, higher severity, and more fatal outcomes with COVID-19 (Hu et al., 2021[70]; Komiyama & Hasegawa, 2020[81]). All these behaviors inhibit the effective immune response to combat COVID-19 and other relevant infections (Figure 4(Fig. 4)).

## Immunocompromised Conditions and COVID-19

The greatest overarching risk factor for COVID-19 infection and negative outcomes is aging (Chen et al., 2021[30]; Manna et al., 2016[98]). Aging brings about many physiological changes such as the deterioration of both innate and adaptive immune responses, the presence of a hyperinflammatory state known as “inflamm-aging”, memory and cognitive impairment, depression, decreased bone and muscle mass, hormonal dysregulation especially that of proper mitochondrial function from the steroidogenic acute regulatory protein and much higher risks for a host of other diseases such as cancers, CVDs, and diabetes (Clegg et al., 2013[33]; Manna et al., 2015[96][97], 2016[98]; Slominski et al., 2013[138]). All such conditions can compound to limit a host's ability to effectively fight off and weather a COVID-19 infection. Furthermore, males have shown to be more susceptible to severe COVID-19 infections and outcomes throughout the aging process (Lewis and Duch, 2021[85]; Peckham et al., 2020[112]). Aging is often accompanied by increased instances of neurological disorders such as Alzheimer's or other dementias can cause physiological effects such as inherent inflammation, increased ACE2 expression, and higher interleukin-6 (IL-6) levels within the bloodstream providing more direct pathways for severe COVID-19 infections. Additionally, decreased cognitive ability can lead to confusion and misunderstanding about proper COVID-19 prevention techniques such as physical distancing and handwashing thus allowing for higher infection rates. Alternatively, uncontrolled and unchecked cell proliferations, i.e. cancers can also lead to higher severe COVID-19 infection rates resulting in hospitalization and death (Dai et al., 2020[38]; Liang et al., 2020[87]). Cancer often leads to other comorbidity development such as CVDs, diabetes, and obesity. Various cancer treatments have shown to significantly reduce a host's immune health and response to a COVID-19 infection (du Plessis et al., 2022[45]; Gosain et al., 2020[60]; Han et al., 2021[65]; Sarfati et al., 2016[127]). Higher rates of cancers are also associated with aging and present added risk and pathways for severe COVID-19 infection. 

Obesity and diabetes present significant risk factors in the progression of COVID-19 and subsequent infections. COVID-19 is known to be adversely affected by an obese condition or one that is characterized by the overabundance and deposition of adipose tissues. Obesity is a known risk factor for a multitude of other deleterious health conditions including diabetes, strokes, cancers, CVDs, liver disease, kidney disease, and mental illness (Mahamat-Saleh et al., 2021[92]; Martinez Steele et al., 2016[99]; Srour et al., 2019[139]). It is usually evolved through the excessive intake of fats and sugars without adequate metabolism, exercise, and intake of proper micronutrients (Sanchis-Gomar et al., 2020[125]). The prolonged and chronic inflammatory state induced from obesity can lead to severe COVID-19 infections and results in more hospitalizations with fatal outcomes. Additionally, the prevention of COVID-19 through vaccination is thought to be inhibited as past studies have shown less CD4+ and CD8+ activation compared to non-obese individuals in trials of other vaccines. Diabetes has shown to induce a proinflammatory state in addition to inhibiting the proper immune cell response in the event of an infection such as COVID-19 (Belikina et al., 2021[11]; Ganesan et al., 2020[56]; Pal and Bhansali, 2020[109]; Sabri et al., 2021[124]). The disease is characterized by either an inability to produce insulin or the development of insulin resistance throughout one's life and is often associated with obesity and poor nutrition. The ACE2 receptor is upregulated in diabetic individuals along with elevated furin serum levels thus increasing the COVID-19 virus' ability to bind with host cells and induce destructive disease progression within the host (Ganesan et al., 2020[56]; Pal and Bhansali, 2020[109]; Sabri et al., 2021[124]). Obesity and diabetes present significant risk factors in the disease progression of COVID-19.

## Assorted Measures for the Prevention of COVID-19 and its Variants

Disease prevention is paramount to modern healthcare, and the COVID-19 pandemic needs special attention as no unique measure is available to control this hostile disease. As a consequence, the WHO, and the governments of numerous countries and their disease prevention and control centers have advocated several actions and/or practices for limiting the spread of COVID-19 infections and its deadly consequences (Ijaz et al., 2021[73]). These preventive procedures include frequent hand washing, sanitization, face coverings, avoidance of parties and/or gatherings, physical distancing, and cleaning of commonly touched surfaces, in addition to available COVID-19 vaccination (Freeman et al., 2014[52]; Manna et al., 2022[95]). Many of these practices also involved shutting down non-essential activities such as travel, school, the workplace, recreation, and meetings/parties (Table 2(Tab. 2); References in Table 2: Anastasi et al., 2020[6]; Avery and Hoffmann, 2018[8]; Bae and Kim, 2020[9]; BourBour et al., 2020[21]; Chazelet and Pacault, 2022[28]; Chu and Wei, 2020[31]; Cooper et al., 2020[37]; Doaei et al., 2021[43]; Freeman et al., 2014[52]; Galmes et al., 2020[55]; Gao et al., 2020[57]; Girum et al., 2021[58]; Gutierrez et al., 2019[64]; Iddir et al., 2020[72]; Ijaz et al., 2021[73]; Lee and Han, 2021[83]; Manna et al., 2022[95]; Pal et al., 2021[108]; Rabie and Curtis, 2006;[117] Rawat et al., 2021[120]; Roncati et al., 2020[121]; Scurr et al., 2022[129]; Shakeri et al., 2022[131]; Shakoor et al., 2021[132]; Shioi et al., 2020[135]; Stach et al., 2021[140]; Steinbrenner et al., 2015[142]; Taha et al., 2021[143]; Taneri et al., 2020[144]; Tang et al., 2021[145]; Tardy et al., 2020[147]; Veys et al., 2021[156]; Vogrig et al., 2021[158]; Wang et al., 2020[163]; Zhang et al., 2020[176]). 

One of these preventive approaches is hand washing, which is known to lower various infectious disease rates, including COVID-19, influenza/seasonal flus, whooping cough, and common cold symptoms caused by viruses/pathogens. It has been reported that a substantial amount of hospital acquired illnesses can be mitigated through the proper hand washing of healthcare workers (Mathur et al., 2011[100]). While hand washing is effective, the COVID-19 virus spreads through aerosol droplets from infected individuals reaching the nose, eyes, and mouth after being expelled through coughing or sneezing. Consequently, the use of face masks has been employed to prevent the spread of COVID-19 (Chazelet and Pacault, 2022[28]; Chu and Wei, 2020[31]). Another protective measure implemented for COVID-19 is physical and social distancing. Studies of viral infections such as influenza have reported that physical distancing of at least one meter is effective at limiting the spread of disease, a scenario certainly influential for protection against COVID-19 infections (Chu and Wei, 2020[31]). Additionally, a number of agents (with pharmacological effects) have been postulated to play fundamental roles in the prophylaxis and/or improvement of COVID-19 associated symptoms. These agents display antioxidative (e.g., vitamin C, trans-resveratrol, kale, and pecans), anti-inflammatory (e.g., miodesin, berries, nuts, and curcumin), and immunomodulatory with either endogenous (e.g., hormones, cytokines, and growth factors) or exogenous (e.g., nutritional supplements such as vitamins, zinc, selenium, and spirulina) effects in preventing and/or ameliorating the severity of COVID-19 allied infections and complications (Ferreira et al., 2020[51]; Zhang et al., 2020[176]). Many of these compounds, including vitamins, phytochemicals, and nutraceuticals, strengthen the immune system for defending against invading pathogens (Table 2(Tab. 2)). Additionally, melatonin, a bioactive compound with many health benefits, along with anti-inflammatory, antioxidative, and immunomodulatory properties, has been reported to regress/limit severe symptoms and complications in COVID-19 patients (Manna et al., 2022[95]; Zhang et al., 2020[176]).

The most effective preventive measure for COVID-19 and other contagious diseases is vaccination/immunization, which fundamentally boosts the immune system, especially in a susceptible population (Roncati et al., 2020[121]; Scurr et al., 2022[129]; Tukhvatulin et al., 2021[152]). Vaccines allow the preemptive development of memory B- and T-cells to neutralize pathogens. However, the efficacies of vaccines are dependent on many factors, including age, immune response disorders, and underlying medical conditions. Three vaccines that are currently available include those developed by Pfizer-BioNTech, Moderna, and Johnson & Johnson in the United States (Singh et al., 2022[136]). These vaccines are highly efficacious, at ages 12 years and older and are capable of reducing the severity of COVID-19 associated complications and hospitalizations. Of note, the Pfizer-BioNTech vaccine is available to individuals ages 5 years and above currently. 

## Therapeutic Potentials for the Treatment of COVID-19 and its Variants

Certain treatment plans have been urgently approved and implemented to manage the severity of COVID-19 associated complications, hospitalizations, and mortalities. Since respiratory complications are commonly observed in COVID patients, the first line of intervention involves artificial oxygen delivery via multiple methods, including noninvasive positive pressure ventilation, intubation and invasive mechanical ventilation, and extracorporeal membrane oxygenation (Boopathi et al., 2021[19]; Hu et al., 2021[71]; Rando et al., 2021[119]). Generally, severe COVID-19 infections are associated with excessive and persistent inflammation in the lungs and other tissues, causing multi organ failure and death. Thus, anti-inflammatory interventions have been introduced in combating COVID-19 complications utilizing different antagonists (Ye et al., 2020[171]). It has been reported that the introduction of mesenchymal stem cells containing the ACE2 receptors into COVID-19 patients suffering from critical illnesses reduces inflammatory responses and disease progression (Leng et al., 2020[84]). Small interfering RNA is another treatment option against COVID-19 patients by introducing RNA molecules that regulate viral gene expression and inhibit future replication (Elekhnawy et al., 2021[47]). 

**Figure 5:**
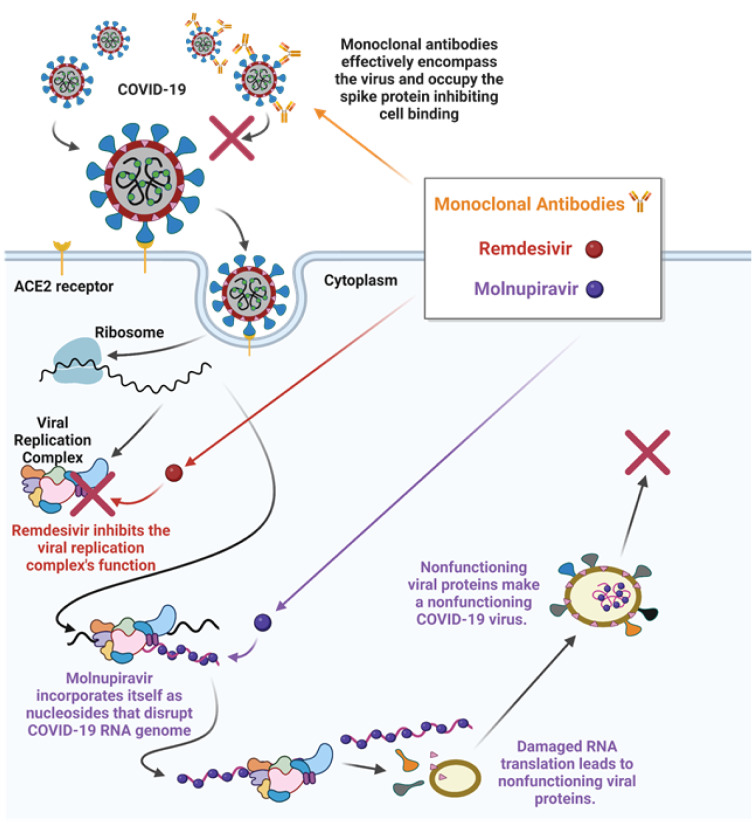
Modes of action of a variety of drugs such as monoclonal antibodies, Remdesivir, and Molnupiravir, against COVID-19. Whereas monoclonal antibodies encompass COVID-19 and occupy the spike protein inhibiting cell binding, Remdesivir block the COVID-19 replication process in a human host cell. Molnupiravir, an oral drug, enters a host cell that is infected by actively replicating COVID-19 viruses, in which it acts as a nucleoside analog that can insert itself into the viral RNA genome. These insertions limit/destroy the original translational function of the genome and lead to the creation of non-functioning COVID-19 proteins that inhibit the virus's ability to replicate and transmit to others. Retrieved and revised from http://app.biorender.com.

The COVID-19 virus uses a number of enzymes, including RNA polymerase, proteases, methyltransferase, and exoribonuclease, for its replication, and this process aligns with SARS and MERS viruses that have been extensively studied (Asselah et al., 2021[7]). Accordingly, several antiviral and antiretroviral drugs being used to combat COVID-19 related complications are based upon other virus treatment regimens (Jin et al., 2020[77]; Kandimalla et al., 2021[79]; Wahid et al., 2021[159]). These drugs include Ribavirin (Tribavirin), Ritonavir (Lopinavir/Norvir), Remdesivir (Veklury), Nelfinavir (Viracept), Umifenovir (Arbidol), and Chloroquine/Hydroxychloroquine (Table 3(Tab. 3); References in Table 3: Al Bishawi et al., 2022[2]; Alavi Darazam et al., 2021[3]; Ambike et al., 2022[5]; Bierle et al., 2021[15]; Bonaventura et al., 2022[17]; Boopathi et al., 2021[19]; Cao et al., 2020[24][25]; Chen et al., 2020[29]; Clemency et al., 2022[34]; Cohen et al., 2021[36]; Davidson et al., 2022[39]; Facente et al., 2021[49]; Guimaraes et al., 2021[61]; Guo et al., 2022[62]; Hosogaya et al., 2021[69]; Hu et al., 2021[71]; Kyriazopoulou et al., 2021[82]; Leng et al., 2020[84]; Liu et al., 2020[88]; Magro et al., 2021[91]; Mahase, 2021[93]; McCreary and Angus, 2020[101]; O'Brien et al., 2021[105]; Origuen et al., 2022[107]; Palanques-Pastor et al., 2020[110]; Peng et al., 2022[114]; Pourkarim et al., 2022[115]; Ramakrishnan et al., 2021[118]; Saber-Ayad et al., 2021[123]; Saravolatz et al., 2022[126]; Singh et al., 2021[137]; Stauffer et al., 2020[141]; Tardif et al., 2021[146]; Tolksdorf et al., 2021[150]; van de Veerdonk et al., 2022[154]; Vargas et al., 2020[155]; Villaescusa et al., 2022[157]; Wang and Yang, 2021[164]; Whitley, 2021[165]; Wise, 2022[166]; Xu et al., 2021[169]; Yu et al., 2021[173]). All of which have been used to treat COVID-19 patients with varying degrees of effectiveness (Cao et al., 2020[24]; Jin et al., 2020[77]). Among these drugs, both Nelfinavir and Lopinavir have worked effectively against previously known SARS and MERS viruses, however, the results are neither satisfactory nor conclusive with COVID-19 (Cao et al., 2020[24]; Yamamoto et al., 2004[170]). Inversely, both Remdesivir and Chloroquine had received EUA by the FDA for treatment of COVID-19 patients, and these drugs, especially Remdesivir, were found to effectively diminish COVID-19 related complications in certain age group individuals (McCreary and Angus, 2020[101]; Wang et al., 2020[161]). Similarly, Umifenovir was reported to have moderate effects in the management of COVID-19 severity (Alavi Darazam et al., 2021[3]). Recently, Merck and Co., in collaboration with Ridgeback Therapeutics, has developed an antiviral oral drug, named Molnupiravir, for the treatment of COVID-19 patients, which shows promising effects against infections and reduces hospitalizations by 50 %. It should be noted that the European Medicines Agency has issued emergency authorization of Molnupiravir (Lagevrio or MK4482) for adult COVID-19 patients suffering with increased complications and illnesses (Pourkarim et al., 2022[115]; Whitley, 2021[165]). Lagevrio has been found to reduce hospitalizations by 31 % and overall risk of death by 89 % when administered within 5 days of the first symptom (Jayk Bernal et al., 2022[75]). Pfizer Inc. has also developed an antiviral oral drug named Paxlovid (Nirmatrelvir/Ritonavir), and this drug therapy (with a low dose of Ritonavir that is used in treating HIV) has been reported to reduce the risk of hospitalizations and mortalities by 89 % when administered within 3 days of the first symptom (Mahase, 2021[93]; Wang and Yang, 2021[164]). While these drugs are effective against COVID-19 and its variants, they act on those viruses differently. For example, Molnupiravir is proposed to function as a nucleoside that incorporates itself into the RNA genome of COVID-19 as it is replicated. The incorporation disrupts the genetic code leading the cell machinery to create COVID-19 proteins with significant errors that no longer function in their proper capacities, thus COVID-19 can no longer reproduce. Remdesivir is a repurposed drug that interacts directly with the viral replication complex to inhibit the replication of the RNA genome and viral proteins blocking the COVID-19 replication cycle. On the other hand, monoclonal antibodies are developed outside of the host organism and they are specific to the COVID-19 virus. Antibodies recognize COVID-19/variants and limit their ability to bind to the host ACE2 receptor. Potential mechanisms of action of antibodies, Remdesivir, and Molnupiravir, for prevention and treatment of COVID-19, have been represented in Figure 5(Fig. 5).

A number of monoclonal antibodies have also been used for the treatment of COVID-19 patients (Table 3(Tab. 3)). These antibodies attach to the spike protein of COVID-19 and limit its ability to bind the ACE2 receptors and allow subsequent replication. The FDA has approved an EUA for antibody-based treatments generated by different pharmaceutical companies, i.e. Bamlanivimab+Estesevimab (Eli Lilly & Co.), Casirivimab+Imdevimab (Regeneron), Sotrovimab (GlaxoSmithKline plc), and Tixagevimab+Cilgavimab (Astrazena), respectively (Cohen et al., 2021[36]; O'Brien et al., 2021[105]; Wise, 2022[166]). A combination of the latter antibodies, called Evusheld, has recently been approved in the United Kingdom for COVID-19 prevention by the Medicines and Healthcare Products Regulatory Agency for immunocompromised people (Wise, 2022[166]). Treatment regimens for these antibodies and/or cocktails include intravenous infusion at the onset of infection to lower the viral load by limiting its initial replication process. Noteworthy, the Regeneron's antibody treatment has also received a sponsorship by the WHO to be used in people who are not developing natural immunity to COVID-19 and may be at a high risk for severe COVID-19 associated illnesses. 

## Summary and Conclusions

COVID-19 is an ever-emerging multi organ system disorder, which represents a serious health crisis all over the world. The manifestations of this disease include aberrant respiratory distress, hypoxia, lung injury, inflammation, and a cytokine storm (Hu et al., 2021[70]; Ye et al., 2020[171]). Patients afflicted with COVID-19 display either mild to moderate, or critical, symptoms with severe complications that involve deadly outcomes in certain cases. Notably, COVID-19 associated morbidities and mortalities are relatively higher with geriatric populations and people with underlying medical conditions such as obesity, diabetes, kidney diseases, autoimmune and inherited diseases, cancers, and neurological disorders, in which the function of immune responses are strikingly impaired (Bohn et al., 2020[16]). Regardless of various immunocompromised situations, men in comparison to women, are drastically more affected by severe COVID-19 associated complications, along with mortalities. As such, maintenance of a strengthened immune system is the primary and natural preventive measure for combating COVID-19 and its genomic variants.

Despite the significance of the immune system, technological advances have provided insight into the molecular events that facilitate a better understanding of COVID-19 pathogenesis. These analyses of COVID-19 have led development of various therapeutic interventions for the management of this severely contagious disease. In accordance with this, a variety of measures have been implemented towards targeting the prevention (e.g. human-to-human transmission) and treatment (e.g. antiviral, antibodies, and others) of COVID-19. Even so, there is no dynamic measure currently available that can effectively prevent and/or treat COVID-19. As emergence of additional patient/clinical data, along with more discoveries, become available, the precise understanding of molecular mechanisms of COVID-19 will lead development of novel therapeutic strategies in the prevention and treatment of this destructive disease.

## Declaration

### Ethical statement

This review article does not include animal or human experiments.

### Acknowledgments

The authors would like to thank many co-workers, collaborators, and the studies of several research groups whose contributions helped in preparing this review article. This work was supported in part by National Institutes of Health grants AG042178, AG047812, NS105473, AG060767, AG069333, AG066347, and R41 AG060836 to P.H.R., and the Department of Internal Medicine to P.R.M.

### Author contributions

Conceptualization, P.R.M.; investigation, P.R.M.; writing-original draft preparation, P.R.M., and Z.C.G.; supervision, P.R.M; writing-review and editing, P.R.M., Z.C.G. M.S., and P.H.R. All authors have read and approved the final version of manuscript for its consideration of publication. 

### Institutional review board statement

Not applicable. 

### Informed consent statement

Not applicable.

### Data availability statement

Not applicable.

### Conflict of interest

The authors declare that there is no conflict of interest that could be perceived as pre-judicing the impartiality of this work.

## Figures and Tables

**Table 1:**
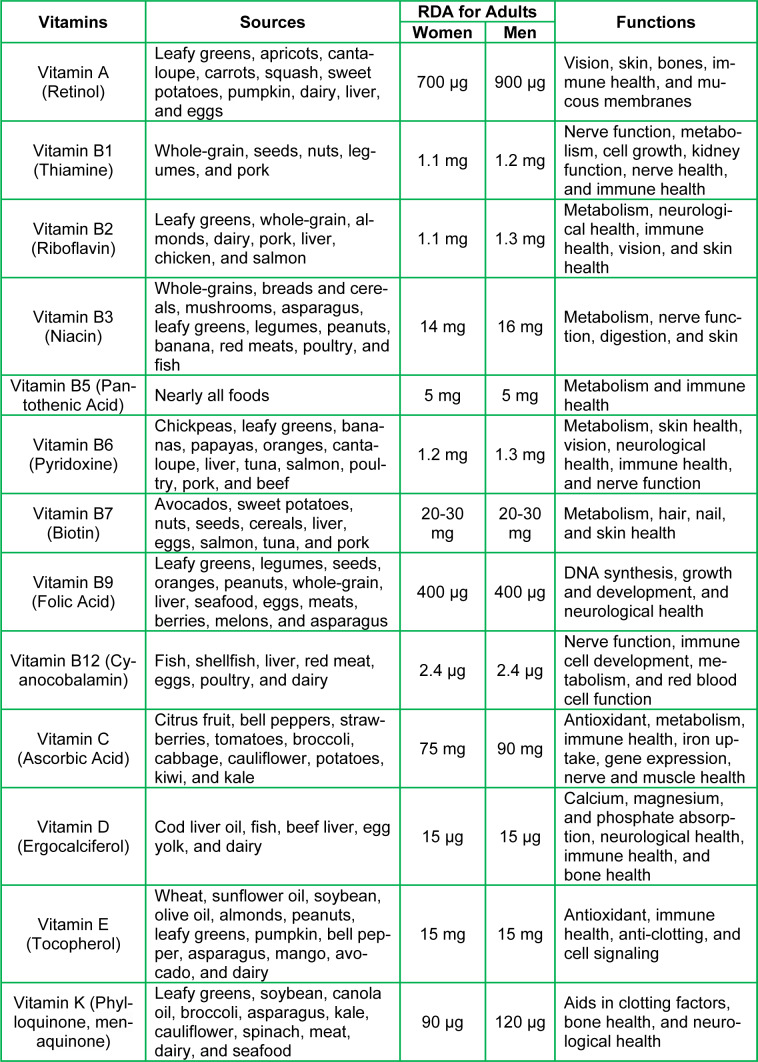
Vitamins, their major sources, RDA values for adults, and various functions

**Table 2:**
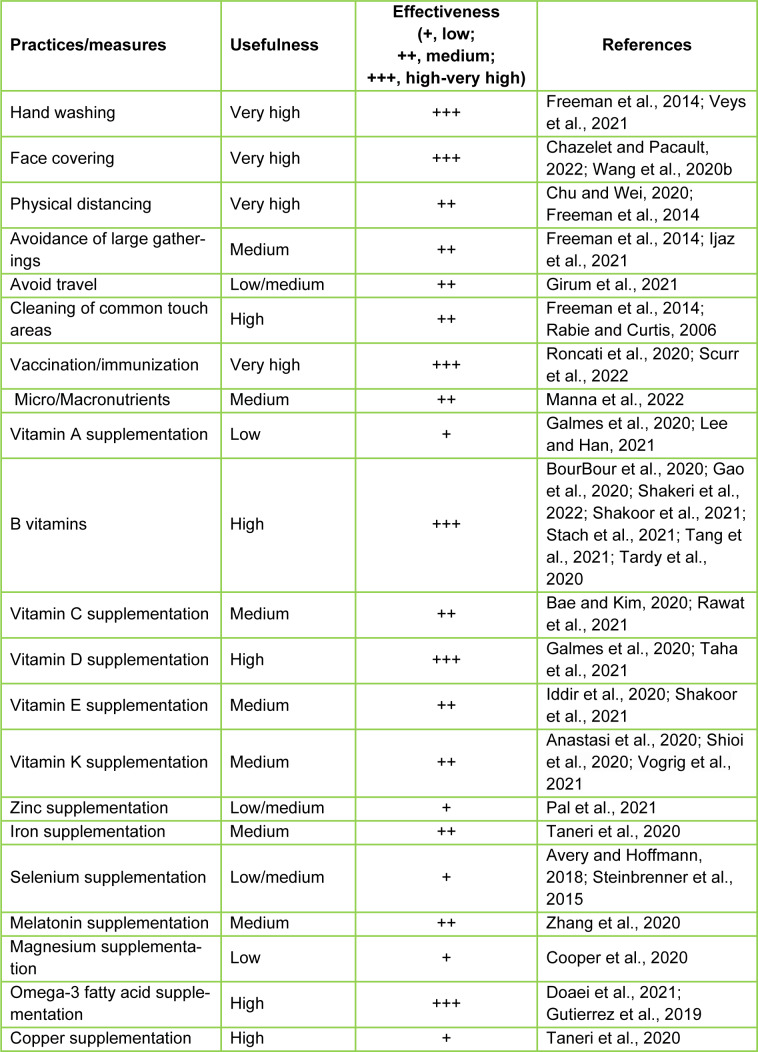
Influence of a variety of practices and/or measures for the prevention of COVID-19 and its variants

**Table 3:**
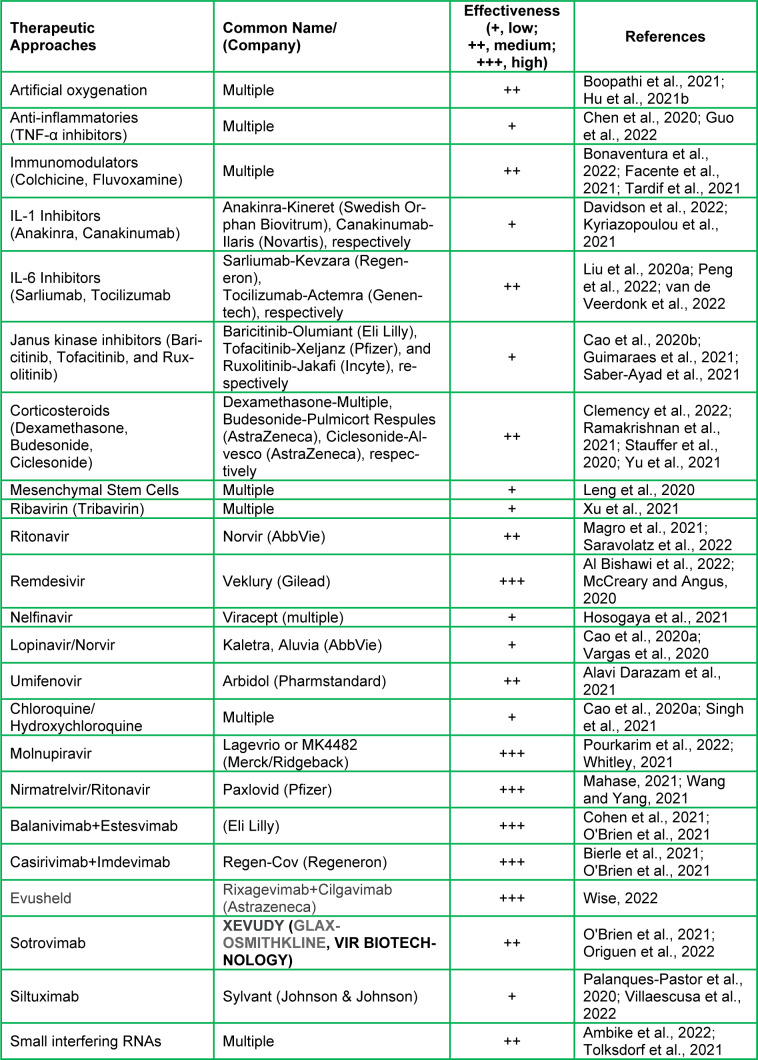
Potential therapeutic interventions for the treatment of COVID-19 and its variants
